# The London Nerve Clinic

**Published:** 1913-11-22

**Authors:** Edwin L. Ash

**Affiliations:** Director. 71 Baker Street, W.


### THE EDITOR'S LETTER-BOX.

### The London Nerve Clinic.

*To the Editor of* The Hospital.

Sir,—To avoid misunderstanding, I shall be extremely obliged if you can grant me a few lines of your valuable space to make it known that the "London Nerve Clinic" (School of Psychotherapy) is in no way connected with any other organisation, institute, or clinic, staffed either by medical or lay workers, in which psychotherapeutic methods are carried out.

Moreover, that although offering treatment on psychological lines to patients of small means, as well as to the very poor, the clinic is entirely independent of outside financial support.—Faithfully yours,

Edwin L. Ash, M.D., Director.

71 Baker Street, W.

